# Supplementary material for: Similarities and differences in waste composition over time and space determined by multivariate distance analyses
Source: PLoS One. 2025 Jan 15;20(1):e0308367. doi: 10.1371/journal.pone.0308367 (PMC11734921; doi:10.1371/journal.pone.0308367)
Supplement: S1 File — (DOCX) [file pone.0308367.s001.docx]

**S1 file.**

**NYC and EPA data use**

**General**

All per capita per year data have been converted to the metric system (kg) (using 2.2 lbs/kg) from tonnage data and reported to three significant figures.

Table S-1 summarizes how the study results were used to generate the six defined waste categories.

| **Study Area** | **NYC** | | | **USEPA** |
| --- | --- | --- | --- | --- |
| **Particular study** | 1990 | 2004 | 2013-2017 |  |
| **Paper** | Paper | Paper | Paper | Paper |
| **Plastics** | Plastics | Plastics | Plastic | Plastics |
| **Glass** | Glass | Glass | Glass | Glass |
| **Metal** | Aluminum  Ferrous  Bimetal cans | Metals | Metal | Ferrous  Aluminum  Other non-ferrous |
| **Food** | Food | Food | Food | Food |
| **Yard Waste** | Grass-Leaves  Brush-Prunings-Stumps | Leaves-Grass  Prunings  Stumps-Limbs | Yard waste | Yard waste |

Table S-1. Category definitions for NYC and EPA studies

**New York City Waste Characterizations**

New York City (NYC) has conducted seven major efforts to define its residential waste stream composition. The 1989 and 2000 efforts were modeling exercises with no field validation. In 1999 physical sampling of the waste stream was done but the scope of the effort was limited to poor recycling areas across the city. The other four, although they differed from each other methodologically, were similar in scope and produced data sets for each borough and for NYC as a whole.

**1990-1991 (“1990 study”)**

The 1990 study sampled residential, commercial, and institutional waste streams although we only extracted the residential data. This study was conducted prior to implementation of the municipal recycling program and so the disposed waste stream was considered to represent all NYC residential waste generation. It was a combined sampling and modeling exercise. NYC was stratified by three income levels (low, medium, high) and three population densities (low, medium, high) creating nine different sampling targets. 23 NYC residential waste routes had ~200 lb (100 kg) samples taken which were then sorted and the results were combined according the stratifications to define each of the nine data classes; additionally, weighting according to each borough’s income and population densities were used to generate borough and NYC-wide compositions, presented as percentages of the waste stream. Four seasonal characterizations (Summer, Fall, and Winter 1990, and Spring 1991) were made as well as an annual characterization. Exactly how the annual characterization was made is not clear; it is not a mean or weighted mean result from the seasonal data; it may be that the seasonal stratified results were combined in some fashion and then applied to overall NYC demographics. In any case, the study had 30 distinct results (four seasons plus an annual value across five boroughs and for NYC as a whole). We reported only the six annual values, as “1990” data, but reported these data as both total waste stream and disposed data. We created per capita per year values from the percentages using annual tonnages given in the report for each borough and the city as a whole plus 1990 US census data for NYC and each borough.

**2004-2005 (“2004 study”)**

The 2004 study sampled residential disposed and recyclable wastes. Recyclables were reported as a single result although NYC was collecting paper recyclables and container recyclables separately (note container recyclables are called “MGP” in NYC, for “metal-glass-plastic”). Exactly how these data were combined for a single recyclables composition was not reported. A combined disposed and recyclables waste stream was also reported, although again how the combined composition of this computed and not directly sampled waste stream was determined was not reported. Composition data were generated similarly to the 1990 study, from sampling of eight income-population strata (low density-low income was eliminated as being poorly represented in New York City) then applying those data sets using borough and NYC-wide representations drawn from the 2000 US census. Four seasonal characterizations (Fall 2004, and Winter, Spring, and Summer 2005) were made as well as a total characterization. This resulted in 90 distinct results (four seasons plus an annual value, five boroughs plus a City-wide representation, disposed wastes and recyclables and a combined total wastes). Again, we did not include the seasonal data, so our percentage data set includes 18 values (the five boroughs and the City-wide value, for total waste stream, disposed wastes, and recyclables). The report included tonnages for each borough for each spring although we used monthly tonnage data and population estimates from the NYC OpenData website to create per capita values. There were differences in sampling methodologies from 1990, particularly in that the 1990 samples included bulk wastes but the 2004 data we report here did not include bulky wastes. The sampling organization, RW Beck, says it identified “pure” collection routes for MSW and recyclables (routes that only covered one of the eight strata) (9,190 routes in total); 3,234 samples were obtained (1,609 MSW, 167 paper only, 684 MGP only, and 774 from split truck routes where both paper and MGP were collected in the same truck, albeit in separate bins). Target sample sizes were 200 lbs (90 kg) for MSW and 100 lbs (45 kg) for recyclables.

**2012-2013 (“2013 study”)**

The 2013 study occurred across two seasons but results were reported as a single annual result for disposed residential wastes, residential recyclables, and a combined waste stream (recyclables again were reported as a single value although DSNY still collected MGP and paper recyclables separately). This resulted in 18 distinct results (five boroughs plus NYC-wide, disposed wastes and recyclables plus a total waste stream estimate). MGP recyclables were sampled in September 2012 and April 2013, paper recyclables were sampled in April 2013, and MSW was sampled September 2012. Trucks to be sampled from each borough (based on DSNY route structures) for the appropriate waste type were selected by random number generation during the sampling period; samples were taken from the truck then transported by the contractor to a central sorting point. This meant the elaborate population density-income stratification was not used this time. The process that the individual samples were combined to create borough and NYC-wide waste descriptions was not explicitly defined, although it seems the mean data from the samples from each borough were used for the borough compositions. The NYC-wide sample was likely to be a weighted sample, either by the relative tonnages or populations for the boroughs; which method was used was not related in the report. A total of 569 samples (256 MSW, 183 MGP, and 130 paper recyclables) were collected as part of the analysis. Target sample sizes again were 200 lbs (90 kg) for MSW and 100 lbs (45 kg) for recyclables.

**2017 (“2017 study”)**

The 2017 study was conducted similarly to the 2013 study, so it resulted in 18 distinct results (five boroughs plus NYC-wide, for total wastes, disposed wastes and recyclables). Organic wastes (779 samples in total) were also sorted separately but were not included in our data because source-separated organics were reported to be 0.4% of the overall 2017 NYC waste stream. The major methodology difference was to create equal-sized samples by filling containers with to-be-sampled wastes until 100 lbs (45.5 kg) (to the nearest lb) had been collected. A total of 581 samples of MSW and recyclables were analyzed (246 MSW samples, 187 MGP recyclables samples, and 148 paper samples). We assume all methods used to generate data sets for 2013 were followed in 2017. Please note the initial report data sheets had incorrect disposal data for Brooklyn; DSNY issued a corrected data set to us in 2023.

**EPA data**

EPA published annual or biannual reports from 1990 to 2020 covering overall US waste composition from 1960 to 2018; the data are not especially structurally comparable to the NYC studies which focused on residential waste streams (EPA reports do not separate the waste stream into generator categories). Each report contains 10 data sets for particular years, and the data may change as the underlying model is altered (so, for instance, EPA has published three different estimates of the tonnage of 1960 disposed paper and six different estimates of the tonnage of 2005 disposed food). Because only 10 data sets are published in each report, some years are not re-reported in later report iterations, while others are. 1990 EPA data were used to cover the 1990 NYC data, 2004 data were used to cover the 2004 NYC data, 2013 data were used to cover the NYC 2013 data, and 2017 data to cover the NYC 2017 data. This means there are 12 EPA data sets (four years for disposed wastes, recyclables, and total wastes). We used national tonnages reported in the specific reports and generated our own per capita per year data using census data for each year. We used the 2017 EPA report for 1990 and 2017 composition data, the 2007 report for 2004 composition data, and the 2014 report for 2013 composition data, as these were the last updated, published model results for each of these years.
