# Supplementary material for: Similarities and differences in waste composition over time and space determined by multivariate distance analyses
Source: PLoS One. 2025 Jan 15;20(1):e0308367. doi: 10.1371/journal.pone.0308367 (PMC11734921; doi:10.1371/journal.pone.0308367)
Supplement: S2 File — (DOCX) [file pone.0308367.s002.docx]

**S2 file.**

**Waste characterizations used in this study:**

Anonymous. Undated. Disposed MSW breakdown (Recyclable and Non-Recyclable) in Washington – based on 1992, 2003 and 2009 waste composition studies (Excel spreadsheet). (Washington 1992, 2009)

Anonymous. Undated. Oregon Tri County Metro 2009: Oregon 2009/2010 waste composition study: Excel results file. (Oregon 2009)

Anonymous. Undated. Orange County waste characterization study – April 2005. Residential waste composition (Exhibit 3-2). (Orange Co. NC 2005)

Anonymous. Undated. Orange County waste characterization study – April 2010. Residential waste composition (Exhibit A-2). (Orange Co. NC 2010)

Anonymous. Undated. Orange County waste characterization study – comparison of residential waste characterizations (Exhibit A-11). (Orange Co. NC 1995, 2000)

Anonymous. Undated. 2017 Pierce County waste characterization results. (Pierce Co WA 2017)

Anonymous. Undated. 2018 Pierce County waste characterization results. (Pierce Co WA 2018)

Anonymous. Undated. 2019 Pierce County waste characterization results. (Pierce Co WA 2019)

Aphale, O., and DJ Tonjes. 2013. Waste generation, waste composition, and differences in source separation in three Town of Brookhaven waste districts. Waste Reduction and Management Institute, School of Marine and Atmospheric Sciences, Stony Brook University. 61 pp. (Brookhaven 2012)

Barton & Loguidice. 2015. Final local solid waste management plan. Monroe County Department of Environmental Services, Rochester, NY. 114 pp. + appendices. (Monroe County 2012)

RW Beck. 2003. Statewide waste characterization study final report. Pennsylvania Department of Environmental Protection. Paged in sections. (Pennsylvania 2001)

RW Beck. 2005. Georgia statewide waste characterization study final report. Georgia Department of Community Affairs. Paged in sections. (Georgia 2003)

RW Beck. 2006. Iowa statewide waste characterization study. Iowa Department of Natural Resources. Paged in sections. (Iowa 2005)

RW Beck. 2009. 2008 Alameda County Waste Characterization Study. StopWaste.org. Pagd in sections. (Alameda Co. CA 2000)

RW Beck. 2010. Multi-seasonal waste characterization analysis. Solid Waste and Recycling, Public Works and Utilities, Pierce County, Washington. Paged in sections. (Pierce Co. WA 1995, 2009)

RW Beck. Undated. NYC waste characterization study (2004-2005). New York City Department of Sanitation. 4 vols. (NYC 2004)

Bureau of Sanitation. 2002. City of Los Aneles waste characterization & quantification study year 2000. Prepared by Cascadia Consulting Group in cooperation with Environmental Science Associates, Sky Valley Associates, Sheri Eiker-Wiles Associates and TerraStat Consulting Group. Solid Resources Citywide Recycling Division, Bureau of Sanitation, Department of Public Works, Los Angeles, CA. 114 pp. (Los Angeles 2002)

CHA. 2014. Solid waste management plan, Capital Region Solid Waste Partnership Planning Unit. Capital Region Solid Waste Partnership Planning Unit, Albany, NY. 2 vols. (Albany 2009)

Camp Dresser & McKee. 2000. Municipal waste composition analysis, final report. Sanitation Division, Department of Streets, City of Philadelphia. Paged in sections. (Philadelphia 1999)

CalRecycle. 2022. 2021 disposal-facility-based waste characterization data tables. CalRecycle, California Department of Resources Recycling and Recovery. 22 pp. (California 2021)

Cascadia Consulting Group. Undated. 2014 residential waste stream composition study final report. Seattle Public Utilities, Seattle, WA. 62 pp. + appendices. (Seattle 2014)

Cascadia Consulting Group. 2004. Statewide waste characterization study. Publication 340-04-005. Contractor’s Report to the Board. Integrated Waste Management Board, State of California. Sacramento, CA. 124 pp. (California 2003)

Cascadia Consulting Group. 2007. 2006 residential waste stream composition study final report. Seattle Public Utilities, Seattle, WA. 52 pp. + appendices. (Seattle 2006)

Cascadia Consulting Group. 2009. California 2008 statewide waste characterization study. Publication #IWMB-2009-023. Contractor’s Report to the Board. California Integrated Waste Management Board, California Environmental Protection Agency, Sacramento, CA. 124 pp. (California 2008)

Cascadia Consulting Group. 2011. 2010 residential waste stream composition study final report. Seattle Public Utilities, Seattle, WA. 62 pp. + appendices. (Seattle 2010)

Cascadia Consulting Group. 2012. 2011 King County waste characterization and customer survey report. King County Waste Monitoring Program, Solid Waste Division, King County Department of Natural Resources and Parks. 114 pp. (King Co WA 2011)

Cascadia Consulting Group. 2015. 2014 disposal-facility-based characterization of solid waste in California. Publication # DRRR-2015-1546. Contractor’s Report. CalRecycle, California Department of Resources Recycling and Recovery, Sacramento, CA. 194 pp. (California 2014)

Cascadia Consulting Group. 2016. 2015 King County waste characterization and customer survey report. King County Waste Monitoring Program, Solid Waste Division, King County Department of Natural Resources and Parks. 122 pp. (King Co WA 2015)

Cascadia Consulting Group. 2020. 2018 disposal-facility-based characterization of solid waste in California. Publication # DRRR-2020-1666. CalRecycle, California Department of Resources Recycling and Recovery, Sacramento, CA. 194 pp. (California 2015)

Cascadia Consulting Group. 2020. 2019 King County waste characterization and customer survey report. King County Waste Monitoring Program, Solid Waste Division, King County Department of Natural Resources and Parks. 119 pp. (King Co WA 2019)

Cascadia Consulting Group. 2021. 2020-2021 Washington statewide waste characterization study. Publication 21-07-026. State of Washington Department of Ecology. (Washington 2020).

Cascadia Consulting Group and the Department of Ecology State of Washington. 2018. Washington statewide waste characterization study. Publication 16-07-032, originally published 2016. State of Washington Department of Ecology. 149 pp. (Washington total and res 2016).

Cascadia Consulting Group and Sky Valley Associates. 2003. 2002 residential waste stream composition study final report. Seattle Public Utilities, Seattle, WA. 52 pp. + appendices. (Seattle 2002)

Cascadia Consulting Group with Sky Valley Associates and RW Beck. 2003. Characterization of waste from single-family residences. City of Phoenix Public Works Department. 20 pp. + appendices. (Phoenix 2003)

Cascadia Consulting Group, Sky Valley Associates, and Sheri Eiker-Wiles Associates. 2000. Waste composition study 1999-2000. Environmental Services Department, City of San Diego. 128 pp. (San Diego 2000)

Cascadia Consulting Group in cooperation with Environmental Science Associates, Sky Valley Associates, Inc., Sheri Eiker-Wiles Associates, and TerraStat Consulting Group. 2002. City of Los Angeles Waste Characterization and Quantification Study Year 2000. Solid resources Citywide Recycling Division, Bureau of Sanitation, Department of Public Works, City of Los Angeles, Los Angeles CA. 114 pp. (Los Angeles 2000)

Cascadia Consulting Group, Sky Valley Associates, and E. Ashley Steel. Undated. 1994/95 residential waste stream composition study final report. Seattle Public Utilities, Seattle, WA. 31 pp. + appendices. (Seattle 1994)

Cascadia Consulting Group, Sky Valley Associates, Sheri Eiker-Wiles Associates, Pacific Waste Consulting Group, Veterans Assistance Network, E. Tseng and Associates, and E. Ashley Steel in cooperation with California Integrated Waste Board staff. 1999. Statewide waste characterization study results and final report. Publication 340-00-009. California Integrated Waste Management Board, Sacramento, CA. 74 pp. + appendices. (California 1999)

Clark County Department of Environmental Services. 2014. The solid waste data report. Appendix J. Solid Waste and Environmental Education Division, Department of Environmental Services, Clark County (WA). 17 pp. (Clark Co. WA 1993, 1995, 1999, 2003, 2008, 2012)

Dvirka and Bartilucci. 1993. Solid Waste Visual Characterization Field Study. Department of Waste management, Town of Brookhaven, Medford, NY. Paged in sections. (Brookhaven 1992).

ESA. 2006. Waste characterization study final report. Department of the Environment, City and County of San Francisco. Paged in sections. (San Francisco 2004)

Environmental Protection Agency. 2015. Advancing Sustainable Materials Management: 2013 Facts and Figures. US Environmental Protection Agency. 65 pp. (EPA 2013)

Environmental Protection Agency. 2019. Advancing Sustainable Materials Management: 2016 and 2017 Tables and Figures. US Environmental Protection Agency. 90 pp. (EPA 1990, 2017)

Green Solutions. 2009. Thurston County waste composition study. Thurston County Solid Waste, Olympia, WA. 36 pp. + appendices. (Thurston Co WA 2008)

Green Solutions. 2014. Thurston County waste composition study. Thurston County Solid Waste, Olympia, WA. 42 pp. + appendices. (Thurston Co WA 2014)

Green Solutions with assistance from Environmental Practices. 2005. 2004 waste composition study final report. Solid Waste Management Division, Department of Water and Waste Management, Thurston County. 36 pp. + appendices. (Thurston Co WA 2004)

Green Solutions with assistance from Environmental Practices and Cascadia Consulting. 2003. Waste composition analysis for the state of Washington. Department of Ecology, Olympia, WA. 15 pp. + appendices. (King Co WA 1999)

Green Solutions with assistance from Skumatz Economic Research Associates. 2000. 1999 waste composition study final report. Solid Waste management Division, Department of Water and Waste Management, Thurston County. 28 pp. + appendices. (Thurston Co WA 1999)

Iowa Department of Natural Resources. Undated. Iowa solid waste characterization. Paged in sections (Iowa 1990, Iowa 1997)

Kessler Consulting Inc. 2017. Orange County waste composition study. Orange County Solid Waste Management, Chapel Hill, NC. 28 pp. + appendices. (Orange Co. NC 2017)

Licata Engineering. undated. Unpublished waste stream categorization in support of a Dirty MRF project, Town of Brookhaven. (Brookhaven 1994)

MSW Consultants. 2011. 2011 Iowa statewide waste characterization study. Prepared for Iowa Department of Natural Resources. Mid-Atlantic Solid Waste Consultants, New market, MD, in conjunction with Cascadia Consulting Group and Foth Infrastructure & Environment. Paged in sections. (Iowa 2011)

MSW Consultants. 2018. Statewide waste composition study. Prepared for Missouri Department of Natural Resources. MSW Consultants, Orlando, FL. Paged in sections. (Missouri 2016)

MSW Consultants. 2020. Onondaga County Resource Recovery Agency 2019 waste characterization study. MSW Consultants, Orlando, FL. Paged in sections. (Onondaga 2019)

The Matrix Management Group, RW Beck and Associates, and Gilmore Research group. 1988. Best management practices analysis for solid waste: Volume One: 1987 recycling and waste stream survey. Publication Np. 88-33A. Office of Waste Reduction and Recycling, Washington State Department of Ecology. 160 pp. (Washington 1987)

The Matrix Management Group and Herrara Environmental, RW Beck, C2S2 Group, and Elway Research. 1991. City of Seattle waste composition study, final report. Solid Waste Utility, Department of Engineering, Seattle, WA. 81 pp. (Seattle 1988, 1990)

Midwest Assistance Program Inc. (The Midwestern Rural Community Assistance Program). Undated. The Missouri Waste Composition Study: Municipal Solid Waste Phase I 1996, Phase II 1997. Missouri Department of Natural Resources. 392 pp. (Missouri 1996, Missouri 1997)

Midwest Assistance Program Inc. (The Midwestern Rural Community Assistance Program). 2007. The 2006-2007 Missouri Municipal Solid Waste Composition Study. Solid waste management Program, Missouri Department of Natural Resources. 392 pp. (Missouri 2006)

Missouri Department of Natural Resources. 1999. 1999: the state of garbage in Missouri. Solid Waste management Program, Division of Environmental Quality, Missouri Department of natural Resources. 44 pp. (Missouri 1987).

NYC Department of Sanitation. Undated. New York City waste composition study (1989-1990). NYC Department of Sanitation, New York, NY. 5 vols. (NYC 1990)

NYC Department of Sanitation. Undated. 2013 NYC Curbside Waste Characterization study. NYC Department of Sanitation, New York, NY. 28 pp + appendix. (NYC 2013)

NYC Department of Sanitation. Undated. 2017 NYC residential, school and NYCHA waste characterization study. NYCsanitation-NYCzerowaste, New York, NY. 65 pp. + on-line spreadsheets. (NYC 2017)

Onondaga County Resource Recovery Agency. 2017. Onondaga County final comprehensive solid waste management plan update. Onondaga County Resource Recovery Agency. 85 pp. +appendices. (Onondaga 1987, 1993, 1998, 2005)

Oregon Department of Environmental Quality. 2004. Oregon solid waste characterization and composition. Contractor: Sky Valley Associates. Department of Environmental Quality, State of Oregon; co-sponsors: Metro; City of Eugene; Marion County. 27 pp. + appendices. (Oregon 1993, Oregon 2000, Oregon 2002)

Oregon DEQ. Undated. Table A-2: Oregon state-wide waste composition 2005: field data and contamination correction (spreadsheet). Posted on DEQ website. (Oregon 2005)

Pierce County Planning and Public Works. Undated. 2016 waste characterization study. Pierce County Planning and Public Works, Pierce County, Washington. Paged in sections. (Pierce Co. WA 2016)

SCS Engineers. 2017. Final 2017 Iowa statewide waste characterization study. Iowa Department of Natural resources, Des Moines, IA. 78 pp. + appendices. (Iowa 2017)

Spendlow, P. 2018. All metro 2016 waste composition study. Excel results files updated June 20, 2018. (Oregon 2016)

Tonjes, DJ, O. Aphale, L. Clark, and KL Thyberg. 2015. Analysis of the change from dual stream to single stream recycling for the Town of Brookhaven (Long Island, NY). Waste Reduction and Management Institute, School of Marine and Atmospheric Sciences, Stony Brook University. 64 pp. (Brookhaven 2014)

US Environmental Protection Agency. 2007. Municipal Solid Waste in the United States: 2006 Facts and Figures. Archived document, US Environmental Protection Agency, associated with EPA-530-F-07-030. 56 pp. (USEPA 2004)
