# Supplementary material for: Similarities and differences in waste composition over time and space determined by multivariate distance analyses
Source: PLoS One. 2025 Jan 15;20(1):e0308367. doi: 10.1371/journal.pone.0308367 (PMC11734921; doi:10.1371/journal.pone.0308367)
Supplement: S3 File — (DOCX) [file pone.0308367.s003.docx]

**S3 file.**

**Notes on waste characterizations used in this study:**

Alameda 2000: reported in Alameda 2008; weighted single family & multi-family per Table ES-3. Data from Table ES-8. Metal single family, multi-family: no white goods reported

Albany NY 2009: Table 4, Appendix B: DGS mean mass fraction. Metals = ferrous metals + non-ferrous metals

California 1999: Table 44; metal no appliances reported; YW = prunings & trimmings + leaves & grass + branches & stumps

California 2003: Table 12: metal no appliances reported; YW = prunings & trimmings + leaves & grass + branches & stumps

California 2008: table 12: metal no appliances reported; YW = prunings & trimmings + leaves & grass + branches & stumps

California 2014: Table 12: metals adjusted for reported appliances; YW = prunings & trimmings + leaves & grass + branches & stumps

California 2018: data used from Table 83 (single family residential) and Table 105 (multi-family residential). Weighted 24:5 based on reported composition of the overall waste stream p.18. metals: no major appliances reported; food = potentially donatable—vegetative + potentially donatable -- eggs, dairy and dairy alternatives + potentially donatable – animal meat + potentially donatable cooked/baked/prepared perishable items + potentially donatable—packaged non-perishable + not donatable – meat + not donatable – non-meat + inedible; = YW = prunings & trimmings + leaves & grass + branches & stumps

California 2021: single family data from Table 2; multi-family data from Table 6. metals: no major appliances reported; food = potentially donatable—vegetative (perishable/fresh) + potentially donatable -- eggs, dairy and dairy alternatives + potentially donatable – animal meat + potentially donatable cooked/baked/prepared perishable items + potentially donatable—packaged non-perishable + not donatable – meat + not donatable – non-meat + inedible; = YW = prunings & trimmings + leaves & grass + branches & stumps

Clark Co WA studies (1993, 1995, 1999); from p. 17; metals adjusted for white goods

Clark Co WA studies (2003, 2008, 2012); from p. 17;

Georgia 2004: data from Table 4-3

Iowa 1990: from Iowa 1997, Table 5 (Comparison of Iowa statewide waste characterization)

Iowa 1997: data from Appendix B Two seasons combined results, Table Iowa all locations residential spring & fall

Iowa 2005: from Table 4-3

Iowa 2011: from Table 3-2

Iowa 2017: from Table 9; food waste = food waste loose + food waste packaged

King Co WA 1999. Reported in Washington 2003, Table 5 (year reported in Table 1). Metals adjusted for white goods.

King Co WA 2011. Table 49; excludes Seattle and Milton

King Co WA 2015: Table 48; excludes Seattle and Milton

King Co WA 2019: Table 47; excludes Seattle and Milton

Los Angeles: weighted average of single family (Table 6, weighted by 863,640 tons) and multi-family (Table 8, weighted by 551,650 tons) data (weights determined by 2000 tonnage data, Table 2 and Table 3). Metal no major appliances reported YW = prunings & trimmings + leaves & grass + branches & stumps

Missouri 1987: presented in Table 1, Missouri Dept of nat res 1999

Missouri 1996. Used Phase I results (Table 24-5). No yard waste results but “other organics = 3.3%” so was comfortable using YW as 3.3% (de minimis error).

Missouri 1997: used Phase II results (Table 24-5). No yard waste results but “other organics = 3.2%” so was comfortable using YW as 3.2% (de minimis error)

Missouri 2006: used Table 1. No yard waste results but “other organics = 2.97%” so was comfortable using YW as 3.0% (de minimis error).

Missouri 2016: total from Table 4-1; res large metro from Table 4-6

Monroe County, NY: Table 7-1, Appendix D. Plastics = PET #1 and HDPE #2 Plastics + Plastic containers #3-#7 + other plastics

Onondaga 1987 (Appendix I, Exhibit 3-4); metals = sum of ferrous and non-ferrous

Onondaga 1993, 1998, 2005. Used resid., adj,. 1993 metals for white goods, metals = ferrous + non-ferrous.

Onondaga 2019. Table A-2.

Orange Co NC 1995/2000 Exhibit A-11 (assumed from 2010): metals = ferrous + non-ferrous

Orange Co NC 2005 Exhibit 3-2 Residential waste composition metals = ferrous + non-ferrous

Orange Co NC 2010 Exhibit A-2 Residential waste characterization metals = ferrous + non-ferrous

Orange Co NC 2016 single family residential: Table 3-4, column “present study” metals = ferrous + non-ferrous

Orange Co NC 2016 multi-family residential: Table 3-5, column “present study” metals = ferrous + non-ferrous

Oregon (metro) 1993; presented in Oregon 2002 (Table A-3); metals adjusted to remove white goods

Oregon 1998: Used Oregon 2002 used field data; Table A-9 (adjusting metals for white goods made no change in data);

Oregon 2000: Used Oregon 2002: used field data; Table A-9 (adjusted metals for white goods);

Oregon 2002: used field data; Table A-5 for metro total (adjusting for white goods made no change in data); Table A-11 for metro res. (no white goods reported)

Oregon 2005: used field data , statewide, Table A-2; adjusted metals for white goods

Oregon 2009 metro res: from spreadsheet tab P09METRES no metals adjustment (no white goods found)

Oregon 2016 metro res: from spreadsheet tab F16METRES; o metals adjustment (no white goods found)

Pennsylvania 2001: Table 3; yard waste = yard waste grass + yard waste other

Philadelphia 1999: Table 3-4. Paper = Corrugated cardboard, newspaper, office paper, magazines, other paper; plastics = PETE bottles and jars, HDPE bottles and jugs, other plastics; Glass = glass food and beverage containers; metal = aluminum cans, other aluminum, steel cans, other ferrous metal, other metal (Not major appliances); yard waste = leaves, other yw.

Phoenix 2003: data from Table 2: YW =Leaves & grass + prunings

Pierce Co WA 1995: reported in Pierce County 2009 Appendix Table Study Statistical Comparison – County-wide, 1995 Waste study vs. 2009 waste study. Adjusted metals for white goods

Pierce Co WA 2009: as reported in Appendix Table Study Statistical Comparison – County-wide, 1995 Waste study vs. 2009 waste study. Did not adjust metals for white goods (none reported)

Pierce Co WA 2016: data from Appendix A, Residential Single family table, residential – multi-family table, and residential self haul table, by weighted average of residential self haul (weighted by 48,000 tons), single family residential (weighted by 130,998 tons) and multi-family units (weighted by 68,089 tons)(weights determined by Table 3-1). Metal res single family, residential self-haul adjusted for white goods; multi-family no white goods reported; food waste = food waste: edible/program compatible, food waste: edible/non-compatible, food waste: non-edible-compatible, food waste: inedible/non-compatible, and k-cups.

Pierce Co WA 2017: Metal res single family adjusted for white goods; multi-family no white goods reported; food waste = food waste: edible/program compatible, food waste: edible/non-compatible, food waste: non-edible-compatible, food waste: inedible/non-compatible, and k-cups.

Pierce Co WA 2018: Metal res single family adjusted for white goods; multi-family no white goods reported; food waste = food waste: edible/program compatible, food waste: edible/non-compatible, food waste: non-edible-compatible, food waste: inedible/non-compatible, and k-cups

Pierce Co WA 2019: Metal res single family, multi-family residential no white goods reported; food waste = food waste: edible/program compatible, food waste: edible/non-compatible, food waste: non-edible-compatible, food waste: inedible/non-compatible, and k-cups

San Francisco (2004): data from Table A-3, a weighted average of single family residential Fantastic 3 (weighted by 129,630 tons) and multi-family residential Fantastic 3 (weighted by 48,082 tons) (weights determined by 2004 rate year tonnage data, Table 3). YW = grass + prunings

Seattle 1989. Fig 1-1. Summed the paper categories.

Seattle 1990. Table III-2. Summed paper, plastics, glass, metals categories. No white goods to adjust for; YW = prunings + leaves and grass

Seattle 1994. Figure 1-1 (p.3) YW = leaves & grass

Seattle 2002. Table 2-2. YW =. prunings + leaves & grass

Seattle 2006. Table 2-2 YW =. prunings + leaves & grass

Seattle 2010. Table 2-2 food = food + fats-oil-grease; YW =. prunings + leaves & grass

Seattle 2014. Table 2-2 food = food + fats-oil-grease; YW =. prunings + leaves & grass

Thurston Co. WA 1999: data from Table 3, a weighted average of residential self haul (weighted by 19,140 tons) and single family homes (weighted by 38,130 tons) (weights determined by 1999 tonnage data, Table 1). Metal res self haul adjusted for white goods reported; single family homes no white goods reported

Thurston Co. WA 2004: data from Table 3, a weighted average of residential self haul (weighted by 21,180 tons) single family homes (weighted by 42,990 tons) and Multi-family units (weighted by 4,340 tons)(weights determined by 2004 tonnage data, Table 1). Metal multi-family adjusted for white goods reported; res self haul and single family homes no white goods reported

Thurston Co. WA 2008: data from Table 3, a weighted average of residential self haul (weighted by 35,648 tons), residential drop-boxes (weighted by 3,341 tons), single family homes (weighted by 37,388 tons) and multi-family units (weighted by 7,990 tons)(weights determined by July 2008-June 2009 tonnage data, Table 2). Metal multi-family adjusted for white goods reported; res self haul, rural drop boxes, and single family homes no white goods reported

Thurston Co. WA 2014: data from Table 4, a weighted average of residential self haul (weighted by 21,490 tons), residential drop-boxes (weighted by 3,435 tons), single family homes (weighted by 46,888 tons) and multi-family units (weighted by 9,686 tons)(weights determined by Sep 2013-Aug 2014 tonnage data, Table 2). Metal rural drop boxes adjusted for white goods reported; res self haul, single family, and multi-family no white goods reported

Washington 1987: Table IV-3. Sum categories by hand. Excluded white goods from metals

Washington 1992, 2009: data presented in tons; percents generated by dividing by sum of waste stream; used summary category classifications

Washington 2016 res: table 12 Food = sum of four food categories (edible food vegetative, inedible food vegetative, edible food meats fats oils, inedible food meats fats oils), Yard waste = sum of 2 YW categories (Yard a& garden Waste leaves and grass, Yard and garden waste prunings)

Washington 2020: Table 18. Metals adjusted for white goods Food = sum of four food categories (edible food vegetative, inedible food vegetative, edible food meats fats oils, inedible food meats fats oils), Yard waste = sum of 2 YW categories (Yard & garden Waste, leaves and grass, Yard and garden waste prunings)
