# Supplementary material for: Similarities and differences in waste composition over time and space determined by multivariate distance analyses
Source: PLoS One. 2025 Jan 15;20(1):e0308367. doi: 10.1371/journal.pone.0308367 (PMC11734921; doi:10.1371/journal.pone.0308367)
Supplement: S4 File — (DOCX) [file pone.0308367.s004.docx]

**S4 file.**

**EPA data sets:**

Environmental Protection Agency. 1975. Resource Recovery and Waste Reduction: Third Report to Congress. EPA/SW-161. Solid Waste Management Office, US Environmental Protection Agency, Cincinnati, OH. 95 pp.

Environmental Protection Agency. 1977. Resource Recovery and Waste Reduction: Fourth Report to Congress. EPA/530-SW-600. Office of Solid Waste, US Environmental Protection Agency, Washington, DC. 142 pp.

JRB Associates. 1981. Solid Waste Data: A Compilation of Statistics on Solid Waste Management Within the United States. EPA Contract No. 68-01-6000. JRB Associates, McLean, VA. 73 pp.

Franklin Associates. 1986. Characterization of Municipal Solid Waste in the United States 1960-2000 (Final Report). PRC Engineering, Chicago, IL. Paged in sections.

Franklin, MA, NS Artz, JE Beachey, VR Sellers, and KL Totten. 1988. Characterization of Municipal Solid Waste in the United States 1960-2000 (Update 1988). EPA/530-SW-88-033. Office of Solid Waste and Emergency Response, US Environmental Protection Agency, Washington, DC. 31 pp.

Environmental Protection Agency. 1990. Characterization of Municipal Solid Waste in the United States: 1990 Update. EPA/530-SW-90-042. Office of Solid Waste and Emergency Response, US Environmental Protection Agency, Washington, DC. 103 pp. + appendix.

Environmental Protection Agency. 1992. Characterization of Municipal Solid Waste in the United States: 1992 Update. EPA/530-R-92-019. Office of Solid Waste and Emergency Response, US Environmental Protection Agency, Washington, DC. Paged in sections, appendices.

Environmental Protection Agency. 1994. Characterization of Municipal Solid Waste in the United States: 1994 Update. EPA/530-R-94-042. Office of Solid Waste and Emergency Response, US Environmental Protection Agency, Washington, DC. 162 pp.

Environmental Protection Agency. 1996. Characterization of Municipal Solid Waste in the United States: 1995 Update. EPA530-R-96-001. Office of Solid Waste and Emergency Response, US Environmental Protection Agency, Washington, DC. 137 pp.

Franklin Associates. 1997. Characterization of Municipal Solid Waste in the United States: 1996 Update. EPA530-R-97-015. Office of Solid Waste and Emergency Response, US Environmental Protection Agency, Washington, DC. 160 pp.

Franklin Associates. 1998. Characterization of Municipal Solid Waste in the United States: 1997 Update. EPA530-R-98-007. Office of Solid Waste and Emergency Response, US Environmental Protection Agency, Washington, DC. 172 pp.

Franklin Associates. 1999. Characterization of Municipal Solid Waste in the United States: 1998 Update. Office of Solid Waste and Emergency Response, US Environmental Protection Agency, Washington, DC. 159 pp.

EPA Archive Document: data tables for the 1998 Update. Unpaged.

Environmental Protection Agency. 2001. Municipal Solid Waste in the United States: 1999 Facts and Figures. EPA530-R-01-014. Office of Solid Waste and Emergency Response, US Environmental Protection Agency, Washington, DC. 136 pp.

Environmental Protection Agency. 2002. Municipal Solid Waste in the United States: 2000 Facts and Figures. EPA530-R-02-001. Office of Solid Waste and Emergency Response, US Environmental Protection Agency, Washington, DC. 165 pp.

Environmental Protection Agency. 2003. Municipal Solid Waste in the United States: 2001 Facts and Figures. EPA530-R-03-011. Office of Solid Waste and Emergency Response, US Environmental Protection Agency, Washington, DC. 170 pp.

Environmental Protection Agency. 2005. Municipal Solid Waste Generation, Recycling and Disposal in the United States: Facts and Figures for 2003. EPA530-F-05-003. Office of Solid Waste and Emergency Response, US Environmental Protection Agency, Washington, DC. 11 pp.

EPA Archive Document: data tables for the Facts and Figures for 2003 report. Unpaged.

Environmental Protection Agency. 2006. Municipal Solid Waste in the United States: 2005 Facts and Figures. EPA530-R-06-011. Office of Solid Waste and Emergency Response, US Environmental Protection Agency, Washington, DC. 153 pp.

Environmental Protection Agency. 2007. Municipal Solid Waste Generation, Recycling, and Disposal in the United States: Facts and Figures for 2006. EPA530-F-07-030. Office of Solid Waste and Emergency Response, US Environmental Protection Agency, Washington, DC. 10 pp.

Data tables for the Facts and Figures for 2006 report. Unpaged.

Environmental Protection Agency. 2008. Municipal Solid Waste in the United States: 2007 Facts and Figures. EPA530-R-08-010. Office of Solid Waste and Emergency Response, US Environmental Protection Agency, Washington, DC. 167 pp.

Environmental Protection Agency. 2009. Municipal Solid Waste Generation, Recycling and Disposal in the United States: Facts and Figures for 2008. EPA530-F-009-021. Office of Solid Waste and Emergency Response, US Environmental Protection Agency, Washington, DC. 10 pp. Attached to USEPA, 2009: MSW Characterization Methodology. 12 pp. and: Municipal Solid Waste Generation, Recycling, and Disposal in the United States: Detailed Tables and Figures for 2008. Unpaged.

Environmental Protection Agency. 2010. Municipal Solid Waste in the United States: 2009 Facts and Figures. EPA530-R-10-012. Office of Solid Waste and Emergency Response, US Environmental Protection Agency, Washington, DC. 189 pp.

EPA Archive Document: US Environmental Protection Agency. 2011. Municipal Solid Waste Generation, Recycling, and Disposal in the United States: Detailed Tables and Figures for 2010. Office of Resource Conservation and Recovery, US Environmental Protection Agency. Unpaged.

Environmental Protection Agency. 2013. Municipal Solid Waste in the United States: 2011 Facts and Figures. EPA530-R-13-001. Office of Solid Waste, US Environmental Protection Agency, Washington, DC. 160 pp.

Environmental Protection Agency. 2014. Municipal Solid Waste Generation, Recycling, and Disposal in the United States: Tables and Figures for 2012. Office of Resource Conservation and Recovery, US Environmental Protection Agency. Unpaged.

Environmental Protection Agency. 2015. Advancing Sustainable Materials Management: 2013 Facts and Figures. EPA530-R-15-002. Office of Resource Conservation and Recovery, US Environmental Protection Agency, Washington, DC. 177 pp.

Environmental Protection Agency. 2016. Advancing Sustainable Materials Management: 2014 Facts and Figures. US Environmental Protection Agency. 65 pp.

Environmental Protection Agency. 2018. Advancing Sustainable Materials Management: 2015 Fact Sheet. EPA530-F-18-004. Office of Land and Emergency Management, US Environmental Protection Agency. 22 pp.

Environmental Protection Agency. 2018. Advancing Sustainable Materials Management: 2015 Tables and Figures. US Environmental Protection Agency. 63 pp.

Environmental Protection Agency. 2019. Advancing Sustainable Materials Management: 2017 Fact Sheet. EPA530-F-19-007. Office of Land and Emergency Management, US Environmental Protection Agency. 21 pp.

Environmental Protection Agency. 2019. Advancing Sustainable Materials Management: 2016 and 2017 Tables and Figures. US Environmental Protection Agency. 90 pp.

Environmental Protection Agency. 2020. Advancing Sustainable Materials Management: 2018 Fact Sheet. EPA530-F-20-009. Office of Land and Emergency Management, US Environmental Protection Agency. 24 pp.

Environmental Protection Agency. 2020. Advancing Sustainable Materials Management: 2018 Tables and Figures. US Environmental Protection Agency. 80 pp.
