# Supplementary material for: Similarities and differences in waste composition over time and space determined by multivariate distance analyses
Source: PLoS One. 2025 Jan 15;20(1):e0308367. doi: 10.1371/journal.pone.0308367 (PMC11734921; doi:10.1371/journal.pone.0308367)
Supplement: S5 File — (DOCX) [file pone.0308367.s005.docx]

**File S5. Comparisons of Euclidean and Manhattan distances for the NYC data**

The relationship between Euclidean and Manhattan distances for the percentage and rate data for the borough-all NYC-EPA data set was investigated (Fig S5-1). There was an overall linear relationship between the two was approximately 2:1. The percentage ratio was 2.1:1 and the rate mean ratio was 2.0:1. Where differences in the data were smaller, the ratios ranged between 1.5 to 2.5, but as the differences between data points increased the ratios clustered more closely around 2.0. In the rate data, the bounding for smaller differences was less well defined, and all of the ratios for larger differences were slightly more than 2.0. The two ratio sets were significantly different (by t-test assuming unequal variances, p <<0.001), and the visual scatter in the ratios and that they differ so for small differences and larger differences indicated that Euclidean and Manhattan distances were not isomorphisms; thus information content generated by these two measures varied. Notably, however, linear regressions for the percentage data and the ratio data returned similar slopes (2.17 for the percentages and 2.19 for the ratios), and the R^2^ value for regressions were greater than 0.95; the large number of values tested apparently offset the clear visual scatter in the data.


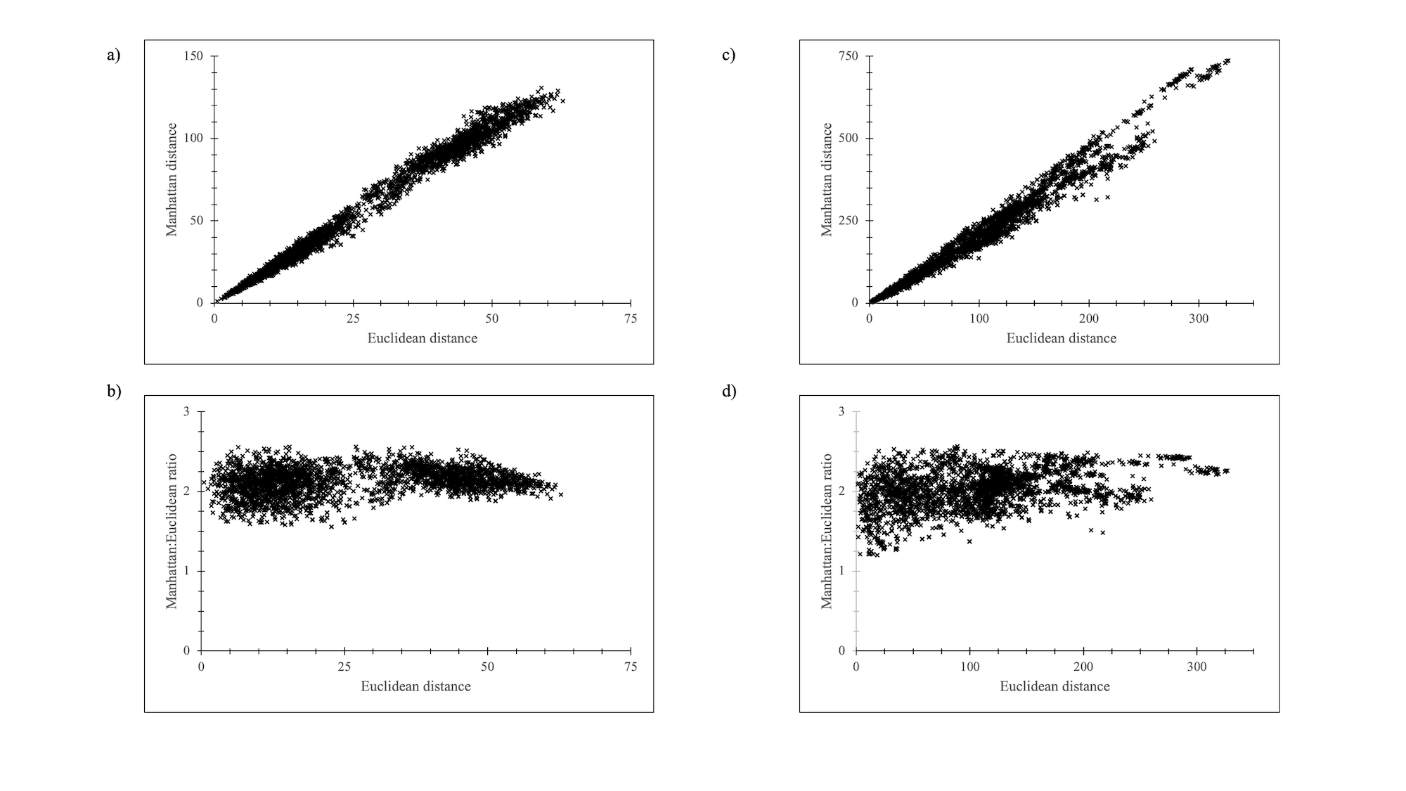


**Fig S5-1. Comparison of Euclidean and Manhattan distances (a-b). Percentage data. (c-d). Rate data**.
