# Supplementary material for: Similarities and differences in waste composition over time and space determined by multivariate distance analyses
Source: PLoS One. 2025 Jan 15;20(1):e0308367. doi: 10.1371/journal.pone.0308367 (PMC11734921; doi:10.1371/journal.pone.0308367)
Supplement: S6 File — (DOCX) [file pone.0308367.s006.docx]

File S6. **Trend graphs for NYC waste generation and disposal rates and EPA disposal rates (reported in [38])**

Figure 5. New York City disposal trends (g/p/d) (by borough) 1992-2020

(solid line = all NYC; x = Bronx; + = Brooklyn; * = Manhattan; ○= Queens; □ = Staten Island)

(drawn from NYC Open Data, https://data.cityofnewyork.us/City-Government/DSNY-Monthly-Tonnage-Data)

NYC total waste trends (g/p/d) (by borough) 1992-2000

(solid line = all NYC; x = Bronx; + = Brooklyn; * = Manhattan; ○= Queens; □ = Staten Island)

(drawn from NYC Open Data, https://data.cityofnewyork.us/City-Government/DSNY-Monthly-Tonnage-Data)

(graph not presented in [38])

EPA disposal data trends g/p/d 1960-2018

Data from EPA reports, last reported datum (per Johnston & Tonjes)

(graph not presented in [38])
