# Supplementary material for: Similarities and differences in waste composition over time and space determined by multivariate distance analyses
Source: PLoS One. 2025 Jan 15;20(1):e0308367. doi: 10.1371/journal.pone.0308367 (PMC11734921; doi:10.1371/journal.pone.0308367)
Supplement: S7 File — (DOCX) [file pone.0308367.s007.docx]

**S7 file. Excel macros useful to extract triangular matrix data**

**Within:**

Sub Euclidian_Within()

Dim i As Integer

Dim rng As Range

Application.ScreenUpdating = False

Worksheets("pcts_euc").Activate

For i = 3 To 29

Set rng = Worksheets("pcts_euc").Range(Cells(2, i), Cells(i - 1, i))

rng.Copy

With Worksheets("Percent_Total").Range("A2")

If IsEmpty(.Value) Then

.PasteSpecial xlPasteValues

Else

.Offset(Rows.Count - .Row).End(xlUp).Offset(1).PasteSpecial xlPasteValues

End If

End With

Next

Application.ScreenUpdating = True

End Sub

**All:**

Sub Euclidian_All()

Dim i As Integer

Dim rng As Range

Application.ScreenUpdating = False

Worksheets("pcts_euc").Activate

For i = 3 To 79

Set rng = Worksheets("pcts_euc").Range(Cells(2, i), Cells(i - 1, i))

rng.Copy

With Worksheets("Percent_Total").Range("D2")

If IsEmpty(.Value) Then

.PasteSpecial xlPasteValues

Else

.Offset(Rows.Count - .Row).End(xlUp).Offset(1).PasteSpecial xlPasteValues

End If

End With

Next

Application.ScreenUpdating = True

End Sub

**Disp:**

Sub Euclidian_Disp()

Dim i As Integer

Dim rng As Range

Application.ScreenUpdating = False

Worksheets("pcts_euc").Activate

For i = 30 To 57

Set rng = Worksheets("pcts_euc").Range(Cells(2, i), Cells(i - 1, i))

rng.Copy

With Worksheets("Percent_Total").Range("G2")

If IsEmpty(.Value) Then

.PasteSpecial xlPasteValues

Else

.Offset(Rows.Count - .Row).End(xlUp).Offset(1).PasteSpecial xlPasteValues

End If

End With

Next

Application.ScreenUpdating = True

End Sub

**Recyc:**

Sub Euclidian_Recyc()

Dim i As Integer

Dim rng As Range

Application.ScreenUpdating = False

Worksheets("pcts_euc").Activate

For i = 58 To 79

Set rng = Worksheets("pcts_euc").Range(Cells(2, i), Cells(i - 1, i))

rng.Copy

With Worksheets("Percent_Total").Range("J2")

If IsEmpty(.Value) Then

.PasteSpecial xlPasteValues

Else

.Offset(Rows.Count - .Row).End(xlUp).Offset(1).PasteSpecial xlPasteValues

End If

End With

Next

Application.ScreenUpdating = True

End Sub
