# Supplementary material for: Similarities and differences in waste composition over time and space determined by multivariate distance analyses
Source: PLoS One. 2025 Jan 15;20(1):e0308367. doi: 10.1371/journal.pone.0308367 (PMC11734921; doi:10.1371/journal.pone.0308367)
Supplement: Attachment — Submitted filename: Response to Reviewers.docx [file pone.0308367.s008.docx]

July 10, 2024

Basant Giri, PhD

Academic Editor

PLOS One

Re: PONE-D-24-14194 revision

Dear Dr. Giri:

The following is our summary of our response to comments on our submission originally titled **Determining differences in waste composition using machine learning techniques** (one comment which we responded to was to change the title). We have attached a table that shows that we responded to all of the reviewer comments, mostly adopting the suggestions they made.

We thank you and the reviewers for the comments that were made. They made it clear to us that we had not clearly presented our thesis. Because of this, we went beyond the scope of the reviewer comments and made more extensive changes to the paper. We think that this product now much better represents what we were trying to achieve. We understand that this may require more substantial review than might have been required if we restricted our changes to the comment set but we think it is better to have made the changes we offer here.

Response to comments table

| Commenter | Number | Global-specific | Comment | Response |
| --- | --- | --- | --- | --- |
| Editor |  | Acknowledgements | Do not include funding statement | Corrected |
|  |  | Data Availability | Minimal data provided | Yes. All data are posted at:  <https://wastedata.info/data-repository>; see the acknowledgement section. |
| Reviewer 1 | 1 | Global | Use common (modern) English: clear, concise, academic tone | Some language choices were made to emphasize approximate nature of waste composition studies; we think too many waste composition studies are overly precise. Thus our initial submission used more generalized terms (a quarter, or “approximately 15%” or “nearly 30%”) because we believe these show there are limits to how exact the measurements from these studies should be reported.  The comment was taken to heart, however, and numerous edits were made to tighten the language usage and use much more precise descriptions of the data (24.4% not “a quarter,” for instance). We decided this was not the battleground to make our stand regarding precision in waste characterizations. We also added more analytical elements to the paper. |
|  | 2 | Global | Uniform font size and style (upper case, lower case) | We revamped tables so that all material is now presented in Times-Roman 12 pt font. Not certain about the concern for upper case-lower case but we tried to be consistent throughout. |
|  | 3 | Title | Rewrite title: clear & informative:  Suggestion: determining spatiotemporal differences in waste generation practices using machine learning techniques | Used: “Similarities and differences in waste composition over time and space determined by multivariate distance analyses” |
|  | 4 | Abstract | More elaborative: explain how study was done w/o methodological details; summarize most important results & significance | The abstract was rewritten to specifically address these points |
|  | 5 | Abstract | No greater than 300 words | Original abstract was 130 words; current abstract is 253 words. |
|  | 6 | Keywords | More suitable: not multivariate, not disposal | Modified: multivariate analyses; disposal trends |
|  | 7 | Keywords | Replace waste studies with waste composition studies | Done |
|  | 8 | Keywords | Eliminate formatical mistake of using lower/upper case in writing key words | Proper nouns were capitalized; common noun were not |
|  | 9 | Introduction | Improve for clarity and conciseness | Efforts were made to achieve this |
|  | 10 | 26-27 | Eliminate parentheses | Parentheses were searched for generally across the paper and mostly removed |
|  | 11 | 27 | Eliminate “many & increasing” | Edited to “causes environmental problems.” |
|  | 12 | 28 | Rewrite “greatest current concern” | Done by combining 27 and 28 |
|  | 13 | Introduction | Provide examples from cited papers | Added: “The value of collected materials for informal recycling is constrained by the metals and plastics content of the wastes [8]; the percentage and energy value of combustible components of the waste stream identifies systems where waste-to-energy incineration is a better option [9]; the organic, readily degradable content of landfilled MSW controls the generation of landfill gas, both as a potential pollutant and as an energy source [10]; and the composition of wastes affects the potential for development of more esoteric energy recovery processes, including pyrolysis, anaerobic digestion, and gasification [11].” |
|  | 14 | Introduction | Eliminate parentheses (& shorten sentences) | Parentheses were search for generally across the paper and mostly removed. |
|  | 15 | 99-146 | Move to materials & methods | Moved part of the material, eliminated much as repetitive. |
|  | 16 | Introduction | Add brief statement of the overall aim of the work & whether that aim was achieved | This was done: “Here we will examine four waste composition sort studies for a particular jurisdiction (New York City) conducted in 1990, 2004, 2013 and 2017. Because these studies of residential wastes not only sorted wastes collected for disposal but also recyclables it was possible to also depict the “generated” residential waste stream, which is not common in these kinds of studies. The percentage and rate data and associated trends for three broad materials categories (paper, food, and plastics) will be compared to EPA data and trends for the same years; the comparisons for disposal will be put into context using 88 other waste composition sort studies from 1988 to 2022. The sort study data trends generally show differences from the EPA data trends.  In addition, we will pioneer a more wholisitc waste stream composition approach that uses the basic techniques that drive machine learning. We will look at the multivariate distance relations among the result sets and use the principle that points close to each other in multivariate space are more similar and points that are farther away are different. This will allow us to underscore several common concepts about overall waste composition for different management processes (disposal and recycling) and to tentatively identify testable theses regarding changes in waste composition over time and space. These results will confirm that EPA modelled composition data tend to be different from composition results generated by physically sorting MSW.” |
|  | 17 | 153 | Justify exclusion of seasonal data | Added: “The seasonal data sets were only available for half of the studies and generated data depiction complications, such as how to show annual values in the context of the four seasonal data points. The multiple closely packed data points across nine months made trend determination unduly complicated.” |
|  | 18 | 172 | Elaborate on 2 non-parametric exceptions | Added: “…using PERMANOVA [Anderson 2005] to determine if significant differences existed across the entire waste stream. The first study determined that there were significant waste stream composition differences across three different waste districts in a Long Island municipality [34]. The second found that waste composition had not significantly changed for those districts two years later (in a broader examination of the impacts of changing from dual stream to single stream recycling) [35].” |
|  | 19 | 616-617 | Description of EPA data are ambiguous | Edited sentences to: As with the NYC percentage data, EPA percentage data increased from 1990 to 2017. Unlike the NYC rate data, EPA rate data also showed increases in food waste generation and disposal. |
|  | 20 | 625 | Specify report cited here | Added another citation to Thyberg et al (ref. 16) |
|  | 21 | Conclusion | Needs to align with aims described at end of the revised Introduction (see comment 16) | 1151-1160 (redline version) original text addressed the first paragraph comment 16. This added material addressed the second paragraph of comment 16:  “It is clear that combining multivariate and standard analyses may improve our understanding of waste composition. The multivariate analyses can show broad patterns of similarity and differences for wastes as a whole based on the principle that points close to each other are similar and points farther way are different. Standard parameter comparisons can help illuminate exactly why the results are different or similar. Thus, disposal processes generate wastes that are different in overall character from recyclables separation. The way the overall character of disposed waste changes over time and distance is different from how recyclables change in character of time and distance. The multivariate analyses confirm that EPA modelled composition data are different in kind from composition results generated by physically sorting MSW.” |
|  | 22 | 749 | Too colloquial “mind you”; maintain academic tone | True; that phrase deleted. |
|  | 23 | Conclusion | End with small paragraph regarding application of study & its future scope: how benefits real world in near future | Done. Added: “Introduction of more wholistic waste stream assessments has the potential for immediate benefits in waste data studies and public planning. Clear identification of similar and different waste composition across jurisdictions adds to understanding of how programmatic factors such as different approaches to collection (Pay-as-You-Throw, single stream recycling) affect the basic units of waste. Being able to quantify the similarities and differences means that more sophisticated determinations of differences across waste systems can be considered. In New York State, where we have a project to assess the waste stream, state planners have created a tool to estimate waste composition based on simple land use classifications (rural, suburban, urban). We can use the wholistic assessments of waste composition to determine the degree that land use helps define waste composition and how other factors may be important, such as basic programmatic differences, or demography, or different approaches to public education. This may help focus and prioritize state efforts to achieve a more circular economy. |
| Reviewer 2 | 1 | Abstract | Too generic: need study novelty, motivation, quantitative information | The abstract was extensively rewritten |
|  | 2 | Introduction | Interconnection between MSW composition and treatment/disposal methods and countries’ legislation | Added: “Generally speaking, developing countries where organic materials especially food wastes are larger proportions of the waste stream tend to rely on landfilling and have less developed formal recycling programs; more developed countries where there may be more fabricated materials in the waste stream tend to have more organized recycling programs and may use incineration with energy recovery as a means of managing wastes. Often governments will specify the management of particular waste streams (such as yard wastes, food wastes, or recyclables) [3].” Ref 3 is What a waste II which contains regional waste management program discussions that support the assertions. |
|  | 3 | Introduction | Explain scope of EPA dataset better | Added: EPA explicitly states that the model is meant to represent the US waste stream as a whole and is unlikely to be representative of MSW at any particular location (EPA 2014). Ref is to EPA methodology report. |
|  | 4 | 99-110 | Move to methods | Eliminated as repetitious, moved following paragraphs into materials and methods and edited the resulting section. |
|  | 5 | 133 | Correct to “one of the largest” | No – it is the largest (2x #2, LA) |
|  | 6 | Introduction | Explain how PCA helps the analysis – any other dimensional reduction strategies can be applied? | This was in the original introduction and was moved to the Materials and Methods section. We think it explains that PCA use was a choice we made and that other dimensional reduction techniques could have been used. We like PCA.  “In addition, statistical analyses such as multidimensional scaling (MDS), principal component analysis (PCA), and various forms of cluster analysis lend themselves to simplified visual depictions of similarities and differences across multivariate data sets such as the waste composition studies. Reducing the dimensionality of multi-dimensional data sets each have relative advantages and issues. Multivariate correlational analyses can also find similarities and differences across these kinds of data sets [33]. Here we use the distance relations and principal component analysis (PCA) to find broader similarities and differences among the four NYC studies and four EPA composition data sets from the same time periods.” |
|  | 7 | General | Detailed descriptive statistics of the entire data set should be provided | Added more descriptive statistics in the Tables and added Tables 5-7. We think this is useful and adds to our work. The original data from the NYC data do and do not have attached descriptive statistics in them (the 1990 report does not but there are pages and pages of data for 2004, 2013, 2017. EPA data do not have descriptive statistics (model outputs). Descriptive statistics are not possible where we combined NYC data sets (because we do not have access to the sample data and the exact means by which the sample data were translated to borough and City-wide data sets. We think simply reproducing the NYC stats is not useful for our analyses. Expanding this effort to cover all of the comparative studies would be a huge effort. We think we have addressed some of the comment but think we do not understand the scope of the effort that this comment is trying to achieve. |
|  | 8 | General | All figures not clear. Improve descriptions of Fig 1-3 | We met the journal figure requirements; we think the figures are clear as we size them (the labels on the heat maps are small but clear). We like our captions for Fig (new numbering) 1, 3 and 4. |
|  | 9 | General | Which plastic is referred to | Plastic here is not a specific plastic but refers to all plastics (containers, films, durable products); see the appendix for materials groups inclusions and exclusions. Added this in Materials and Methods: “The categories include all subcategories (if any) of the waste sorts. So “plastics” here includes all containers of various resin types, plastic films, and all durable plastics.” |
| Reviewer 3 |  | Introduction | Revamp: paragraphs not well connected | The introduction was extensively rewritten. |
|  |  | Results | Highlight main observation (too many details) | Worked hard to make the focus better |
|  |  | General | Trend prediction possible? | Not our intention to make predictions |
|  |  | 61 | Mentions neural networks but do not include such results | We wanted to acknowledge that some are using this technique; we are not (yet) impressed with the results. |
|  |  | 99 | Analyses of the 214 studies done? | No, and deleted this reference. |
|  |  | 108-110 | Promise of future release not appropriate here | Deleted this section |
|  |  | Methods | Highlight the seven parameters again (lost from introduction) | Done |
|  |  | 236-239 | Overall waste generation rates: which figure? | They are in ref 38. Added relevant figures in File S6 |
|  |  | Figures 5-9 | Improve captions (more descriptive) | We think the captions are adequate. |
|  |  | Results | Too cluttered with details | Agreed; we simplified the discussions. |
|  |  | Results | Why the section on the correlation between Manhattan and Euclidean distances | Agreed. It was removed. |
|  |  | Title | Not appropriate; results analyzed by std. stats. Machine learning raises expectation of predictive tools; is this intended to be predictive (if so, provide an example) | We think of machine learning as a grouping process: it is used to determine whether things are similar or different, at the very basic level. The algorithms for doing this are often hidden due to the process used to determine these similarities. For applications like Chat GPT, the models generate members of a class that can be anticipated to follow on a particular prompt; we don’t think this “predictive.” Here we would use the grouping process to determine if a set of waste composition data was similar to (close to) or different (far from) another result or set of results. We think of this as a classification process not predictions.  But the point is taken. We have removed (nearly) all references to machine learning |
|  |  | 14 | Found should be revealed/showed | Substituted showed |
|  |  | 15 | More context on data trends upfront (comp to 88 other studies) | Abstract was revised to include more details |
|  |  | 17 | Not novel; commonly used | Disagree; multivariate distance measures commonly used in many fields but never before in waste composition studies |
|  |  | 19 | Specify conclusions that were reached | Abstract was revised to include more details. |
|  |  | 20 | Context of “time & distance” | Specified that they applied to the NYC data sets |
|  |  | 27 | Many & increasing: many increasing | Deleted the clause |
|  |  | 31 | However: eliminate | Done |
|  |  | 34: | US is an exemplar: An example of such a case is the US | Removed the sentence. |
|  |  | 45 | Expand about the cited paper | This is covered in the discussion; would be repetitive to do it here too. |
|  |  | 76-79 | Explain derivation of rates better | Added: “That is, given a percentage value for a particular material, the amount of that material can be determined by multiplying the percentage by the total amount of wastes. Dividing the resultant amount of wastes by the relevant population and a time measure, such as 365 for a daily value, will create a rate datum.” |
|  |  | 80-81 | Not clear (extraction of data across studies) | Edited to: “Thus, it is possible to collect a set of waste composition studies, determine if the procedures used to generate the data sets were congruent (the data are for disposal, or are all residential wastes, etc.), and then use the data sets in various combinations to extend the relevance of the studies.” |
|  |  | 89-90 | Move however to front of clause | Done |
|  |  | 199-201 | Not clear (Manhattan distance explanation) | Sentence removed; we no longer discuss Manhattan distances. There is a brief, reconsidered discussion in the materials and methods section |
|  |  | 277-278 | Not clear | Agreed; added the trend for plastics rates “was flat.” |
|  |  | 442-444 | Truer is not justified here | Substituted: “more directly measure differences for” for “are a truer depiction” |

We believe that we have addressed the reviewer concerns, as documented above. We know that we have improved the paper considerably under their guidance and in reaction to their comments. We believe that this is a much better paper.

We understand if the scope of changes we made cause another round of review, although we hope this is not the case.

Yours truly,

David J. Tonjes

Corresponding Author
